# DNA insecticide developed from the *Lymantria dispar* 5.8S ribosomal RNA gene provides a novel biotechnology for plant protection

**DOI:** 10.1038/s41598-019-42688-8

**Published:** 2019-04-17

**Authors:** Volodymyr V. Oberemok, Kateryna V. Laikova, Nikita V. Gal’chinsky, Refat Z. Useinov, Ilya A. Novikov, Zenure Z. Temirova, Maksym N. Shumskykh, Alisa M. Krasnodubets, Anna I. Repetskaya, Valeriy V. Dyadichev, Iryna I. Fomochkina, Evgenia Y. Bessalova, Tatiana P. Makalish, Yuri I. Gninenko, Anatoly V. Kubyshkin

**Affiliations:** 1 Department of Biochemistry, Taurida Academy, V.I. Vernadsky Crimean Federal University, Vernadsky Avenue 4, 295007 Simferopol, Crimea Ukraine; 2 Medical Academy named after S.I. Georgievsky, V.I. Vernadsky Crimean Federal University, Lenin Avenue 5/7, 295051 Simferopol, Crimea Ukraine; 3 Botanical Garden named after N.V. Bagrov, V.I. Vernadsky Crimean Federal University, Vernadsky Avenue 4, 295007 Simferopol, Crimea Ukraine; 4 Engineering Center, V.I. Vernadsky Crimean Federal University, Vernadsky Avenue 4, 295007 Simferopol, Crimea Ukraine; 5 All-Russian Research Institute for Silviculture and Mechanization of Forestry, Institutskaya Street 15, 141200 Pushkino, Russia

**Keywords:** DNA, Model invertebrates, PCR-based techniques

## Abstract

Having observed how botanicals and other natural compounds are used by nature to control pests in the environment, we began investigating natural polymers, DNA and RNA, as promising tools for insect pest management. Over the last decade, unmodified short antisense DNA oligonucleotides have shown a clear potential for use as insecticides. Our research has concentrated mainly on *Lymantria dispar* larvae using an antisense oligoRING sequence from its inhibitor-of-apoptosis gene. In this article, we propose a novel biotechnology to protect plants from insect pests using DNA insecticide with improved insecticidal activity based on a new antisense oligoRIBO-11 sequence from the 5.8S ribosomal RNA gene. This investigational oligoRIBO-11 insecticide causes higher mortality among both *L. dispar* larvae grown in the lab and those collected from the forest; in addition, it is more affordable and faster acting, which makes it a prospective candidate for use in the development of a ready-to-use preparation.

## Introduction

As the approaches used to develop preparations to protect plants from insect degradation have evolved, a pivotal moment has arrived: the beginning of the end of the hegemony held by chemical insecticides as they are replaced by better, more efficient preparations based on nucleic acids. Chemical insecticides (neonicotinoids, pyrethroids, organophosphates, carbamates, anthranilamides, etc.) are affordable and fast acting, but they have long half-lives, potentially serious environmental consequences, and are non-selective. While biological preparations are selective, they are slow acting and relatively expensive to produce. DNA insecticides and RNA preparations are inherently chemical insecticides made of natural polymers that, due to the principle of complementarity, act in a highly selective manner. Thus, DNA insecticides and RNA preparations are able to unite the best characteristics of the other types of modern insecticides, without the disadvantages, and can be synthesized in huge quantities on automatic equipment.

We have been investigating DNA-based insecticides for more than a decade. In the forested areas adjacent to our urban areas, gypsy moth (*Lymantria dispar* L.) causes enormous amounts of damage to hundreds of plant species^1,2^. The larvae are voracious feeders, able to consume more than 1 m^2^ of foliage per larva during the caterpillar stage. During outbreaks, when the populations surge, larvae are capable of completely defoliating host trees and decimating cereal crops and vegetables growing near forested areas. Gypsy moth populations can reach outbreak levels in many areas of the world, causing economic losses to forests in Europe, Asia, Africa, North America^3^, and even New Zealand^4^. This led us to investigate how to create DNA-based insecticides to help address this damage^5–7^, because phytophagous insects, such as the gypsy moth, present an ideal insect for testing topical application of insecticides produced from nucleic acids^8^.

Our investigation of unmodified antisense DNA oligonucleotides developed from the RING (really interesting new gene) domain of the LdMNPV (*Lymantria dispar* multiple nucleopolyhedrovirus) IAP-3 (inhibitor-of-apoptosis) gene has recently shown that these oligonucleotides can cause high mortality among LdMNPV-free larvae^9^ and LdMNPV-infected gypsy moth larvae^5,10^. The oligoRING (5′-CGA CGT GGT GGC ACG GCG-3′), acting as an antisense RNase H-dependent oligonucleotide, induces the degradation of target mRNA for the host IAP-Z gene (very homologous to LdMNPV IAP-3 gene) in LdMNPV-free larvae. This is followed by downregulated expression of the target protein. Data collected with respect to insect mortality, the accumulation of biomass, and histology suggest that the oligoRING induces apoptosis in LdMNPV-free larval insect cells. Unfortunately, some LdMNPV-free larvae reared from egg masses under laboratory conditions failed to show any significant sensitivity to the oligoRING fragment^10^. After further investigation, we believe that the insecticidal effect caused by the oligoRING on LdMNPV-free gypsy moth larvae results from interference with host IAP-Z gene expression. This leads to cell apoptosis and larval death. The insecticidal effect of the oligoRING is dependent upon the relationship between the synthesis and the breakdown of the target host’s mRNA IAP-Z gene. The observation that healthy LdMNPV-free gypsy moth larvae grown under lab conditions are not affected by the oligoRING, unlike larvae exposed to stress factors^6,9^ in natural habitats, are similar to those of researchers examining the effects of RNAi in lepidopterans^11^, and provides an explanation why the oligoRING does not always exert its insecticidal effect. In a complex natural habitat, such as a forest, the presence of many stress factors (baculoviruses and bacterial infections, UV radiation, air pollution, etc.) may activate an apoptosis–anti-apoptosis system^10,12^. This provides compelling evidence explaining why earlier-stage larvae collected from forests experienced significant mortality as the result of the down-regulation of the target host IAP gene, unlike LdMNPV-free larvae grown under laboratory conditions. We believe that the use of insecticides developed from the oligoRING to control gypsy moth predation will perform much better under natural conditions in forests^10^. However, we decided to tackle the abovementioned problem by inducing significant insecticidal mortality in *L. dispar* larvae grown in the lab and resistant to the oligoRING insecticide. We decided to include ribosomal RNA (rRNA) as another target for DNA insecticide development. While mRNA comprises just 5% of cellular RNA, rRNA, being more abundant, accounts for 80% of cellular RNA and is metabolically stable^13,14^, making rRNA an excellent candidate for this purpose.

We chose the 5.8S rRNA gene for these experiments since successful application of its fragments as antisense oligonucleotides is well documented. Studies of the inhibition of protein synthesis by specific anti 5.8S rRNA oligonucleotides have suggested that this RNA plays an important role in eukaryotic ribosome function. Speculation regarding the function of this sequence has focused on whether it plays a role in tRNA binding^15,16^ and ribosome translocation^17^, at least in a universally conserved GAAC sequence region common to all 5.8S RNAs^18^, since the region surrounding it is often species specific^15^. Significant and reproducible inhibitions with several different unmodified DNA oligonucleotides have been produced using rabbit reticulocytes; among these, the most inhibitory were specific for the universally conserved 5′-GAAC-3′ sequence. In the experiments carried out using rabbit reticulocyte extract, maximum inhibition was observed when an oligomer 10–11 nucleotides long was used; longer chain lengths resulted in reduced inhibition. A similar reduction was observed with wheat germ extract. With each type of extract, mutated sequences (even single-nucleotide mutations) significantly reduced the level of inhibition^19^, providing a basis for selectivity of action. As a result, we designed an 11 nucleotide long (5′-TGCGTTCGAAA-3′) antisense oligonucleotide (oligoRIBO-11) from the *L. dispar* 5.8S ribosomal RNA gene that includes the universally conserved antisense 5′-GTTC-3′ sequence and applied it as a contact DNA insecticide in our experiments.

## Results

### Mortality of *L. dispar* larvae grown in the lab

**Figure 1:**
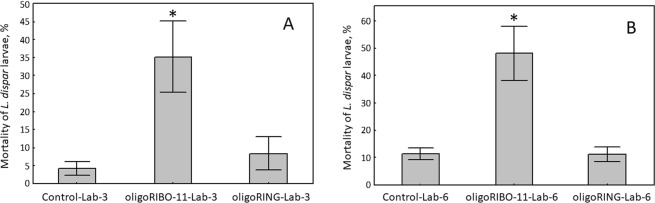
The effect of oligoRIBO-11on the mortality of gypsy moth larvae grown under lab conditions and resistant to oligoRING insecticide: (**A**) 3 days after the treatment; (**B**) 6 days after the treatment. *Indicates results with a significant difference compared with those of the Control (p < 0.01). SE (standard errors) are given for five replicates.

In *L. dispar* larvae grown in the lab and resistant to oligoRING insecticide, insect mortality increased significantly on the 3^rd^ day after treatment in the oligoRIBO-11 group (χ^2^ = 28.7, p < 0.01) compared with the mortality of larvae in the control (water-treated) group (Fig. 1A). In the groups treated with oligoRIBO-11, oligoRING, and water, we observed larval deaths of 35.3%, 8.3%, and 4.2%, respectively. On the 6^th^ day after treatment, we observed a statistically significant increase in insect mortality caused by oligoRIBO-11 compared to mortality seen in the control (water-treated) group (χ^2^ = 31.2, p < 0.01). Among the three treatments, dead insects were found in the following percentages: 48.1% (oligoRIBO-11), 11.4% (oligoRING), and 11.1% (water), respectively (Fig. 1B). Thus, for the first time, we were able to significantly increase mortality in lab-grown, oligoRING insecticide-resistant *L. dispar* larvae using another contact DNA insecticide (oligoRIBO-11).

Lab-grown *G. mellonella* larvae (a non-target lepidopteran) were treated with oligoRIBO-11 and oligoRING, followed by 6 days of observation. During that time, there was no statistically significant effect (p > 0.05) on the viability of the larvae compared with that of water-treated controls. All experimental groups had a mortality rate of 0%. This demonstrates that DNA insecticides are selective and that their specificity of action depends on the particular combination of nitrogenous bases in a fragment, as shown previously in other non-target insects^5,20^. This is explained by the fact that this non-target lepidopteran has a section of 5.8S ribosomal RNA (5′-CTTCGAACGCA-3′) that is not completely complementary to that of oligoRIBO-11. We discovered this in the course of additional research.

### Mortality of *L. dispar* larvae collected from a natural habitat

We also tested oligoRIBO-11 insecticide on *L. dispar* larvae grown in a natural habitat (pubescent oak forest, *Q. pubescens*). Mortality assessed 3 days after treatment showed significantly increased mortality for the *L. dispar* larvae, both in the oligoRIBO-11 group (χ^2^ = 30.3, p < 0.01) and in the oligoRING group (χ^2^ = 23.8, p < 0.01) compared with mortality in the control (water-treated) group (Table 1).

**Table 1:** Mortality (%) of *L. dispar* larvae.

Day	Control-Forest	oligoRIBO-11-Forest	oligoRING-Forest
3^rd^	0	28.3 ± 5.2*	23.4 ± 8.7*
6^th^	0	46.9 ± 9.3*	25.3 ± 8.2*

The significant difference compared with results from the Control group is indicated by * for p < 0.01. SE (standard errors) are given for five replicates.

On average, 28.3%, 23.4%, and 0% of the larvae died in the groups treated with oligoRIBO-11, oligoRING, and water, respectively. On the 6^th^ day after treatment, the increased mortality among insects treated with oligoRIBO-11 and oligoRING was statistically significant compared to that of the controls (water-treated) (χ^2^ = 58.9, p < 0.01 for oligoRIBO-11 vs. Control and χ^2^ = 26.3; p < 0.01 for oligoRING vs. Control); the percentage of dead insects reached 46.9% (oligoRIBO-11), 25.3% (oligoRING), and 0% (water), respectively (Table 1). Both of the DNA insecticides, oligoRING and oligoRIBO-11, were significantly effective against *L. dispar* larvae grown in their natural habitat. Of note, oligoRIBO-11 was 1.85 times more effective as an insecticide than oligoRING and eliminated about half of the insect pests within 6 days. Thus, in contrast to the oligoRING insecticide, the oligoRIBO-11 insecticide is more effective^10^, faster in action^9^, and more affordable (shorter than the oligoRING by 38.9%), which makes it an excellent candidate for development into a ready-to-use preparation.

One of the most important aspects of insect pest control involves getting a significant amount of the preparation into the environment, which is frequently a large area that may feature difficult terrain. Using unmodified antisense oligonucleotides as natural oligomers seems to be the safest way to do this, since cells contain ubiquitous nucleases that can neutralize them. The longest lasting insecticidal effect occurs only when an insect pest contains the RNA of the target gene. It is important to investigate the activity of intracellular nucleases in both *L. dispar* and *Q. pubescens*, a major host plant for the gypsy moth in Crimea, and evaluate the half-life and biodegradability of DNA insecticides in target and non-target organisms.

**Figure 2:**
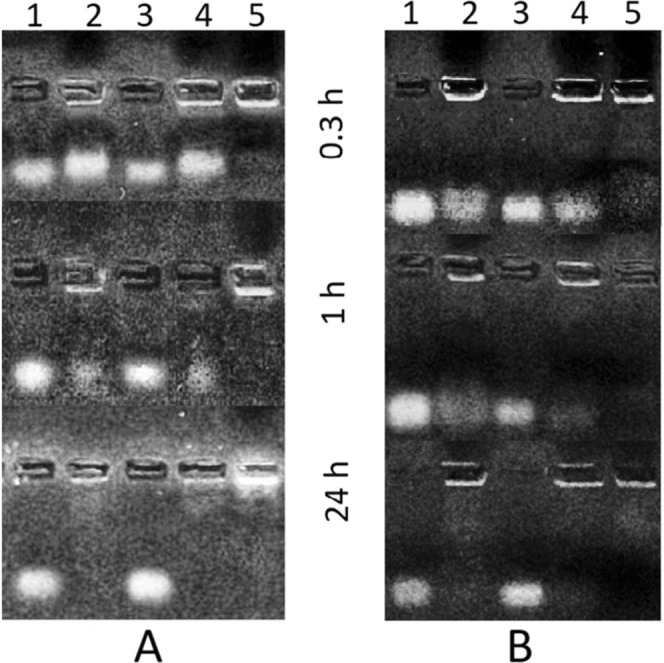
Electrophoregram (1.8% agarose gel) representing the activity of intracellular nucleases of *L. dispar* (**A**) and *Q. pubescens* (**B**) after 0.3, 1, and 24 hours at 27 °C: 1 – control (10 μL of oligoRIBO-11 at a concentration of 150 pmol/μL); 2 – tissue homogenate (1.5 mg of biomass per 10 μL of distilled water) + 10 μL of oligoRIBO-11 at a concentration of 150 pmol/μL; 3 – control (10 μL of oligoRING at a concentration of 5 pmol/μL); 4 – tissue homogenate (1.5 mg of biomass per 10 μL of distilled water) + 10 μL of oligoRING at a concentration of 5 pmol/μL; 5 – pure tissue homogenate (1.5 mg of biomass per 10 μL of distilled water).

Our data showed high activity for the intracellular nucleases of *L. dispar* and *Q. pubescens*, which completely degraded oligoRIBO-11 and oligoRING fragments within 24 hours (Fig. 2). In general, it is assumed that during treatment of crops, only about 0.1% of the insecticides reach insect pests^21^, since 99.9% of insecticide applications miss their targets and enter the soil, water reservoirs, and the atmosphere. All of these habitats are home to organisms containing ubiquitous nucleases that can neutralize the oligonucleotides.

In contrast to chemical insecticides, DNA insecticides will not accumulate in the tissues of target and non-target organisms, and, subsequently, in the surrounding environment. In addition, they are effective against a particular insect pest and implement the principle of highly selective insecticides – an urgent need for agriculture and forestry.

### The oligoRIBO-11 insecticide significantly decreases the concentration of the 5.8S mRNA in *L. dispar* larvae

**Figure 3:**
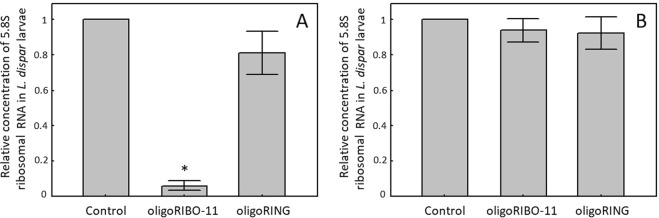
Relative concentration of 5.8S ribosomal RNA in *L. dispar* larvae 6 (**A**) and 12 (**B**) days after treatment with the oligoDNAs. Data represent the means and standard errors of ribosomal RNA concentrations for 3 replicates relative to the control (water-treated) group. *Indicates a significant difference between the data for the oligoRIBO-11 group and those for the control (water-treated) group when p < 0.01.

Much evidence exists that the mechanisms by which DNA insecticides function are similar to those of oligonucleotides used in medicine, both unmodified^22^ and modified antisense^23^. The antisense effects of DNA insecticides are generated using an RNase H-dependent mechanism^24,25^. The RNA strand of an RNA/DNA duplex is hydrolyzed by the enzyme RNase H. For this reason, on the 6^th^ day after treatment with the oligoDNAs, we decided to check the relative concentration of 5.8S ribosomal RNA in *L. dispar* larvae grown in the lab. Several studies have determined that the 5.8S ribosomal RNA represents the most stable reference genes^26,27^, providing an accurate and reproducible real-time PCR assay. 28S and 5.8S rRNAs constitute about 85–90% of total cellular RNA, and are very useful as internal controls^28^. The concentration of the 5.8S ribosomal RNA in oligoRIBO-11-treated insects was significantly lower (16.5 fold) compared with that of the controls (water-treated) (p < 0.01, Fig. 3). The ribosomal subunit 5.8S RNA is an essential part of the protein synthesis complex. A 94% decrease in its concentration was efficiently carried out by oligoRIBO-11-assisted RNase H-dependent reduction, which significantly increased the mortality of oligoRIBO-11-treated larvae. In contrast, the effect of treatment with the oligoRING on the decreased concentration of 5.8S ribosomal RNA in *L. dispar* larvae compared with that of the controls (water-treated) was not statistically significant (p > 0.05). Thus, the mechanism of action of antisense oligoRIBO-11 is specific; we believe it to be the same mechanism used by medical antisense RNase H-dependent oligonucleotides^22^ and the oligonucleotides used for *L. dispar* control^10^.

**Figure 4:**
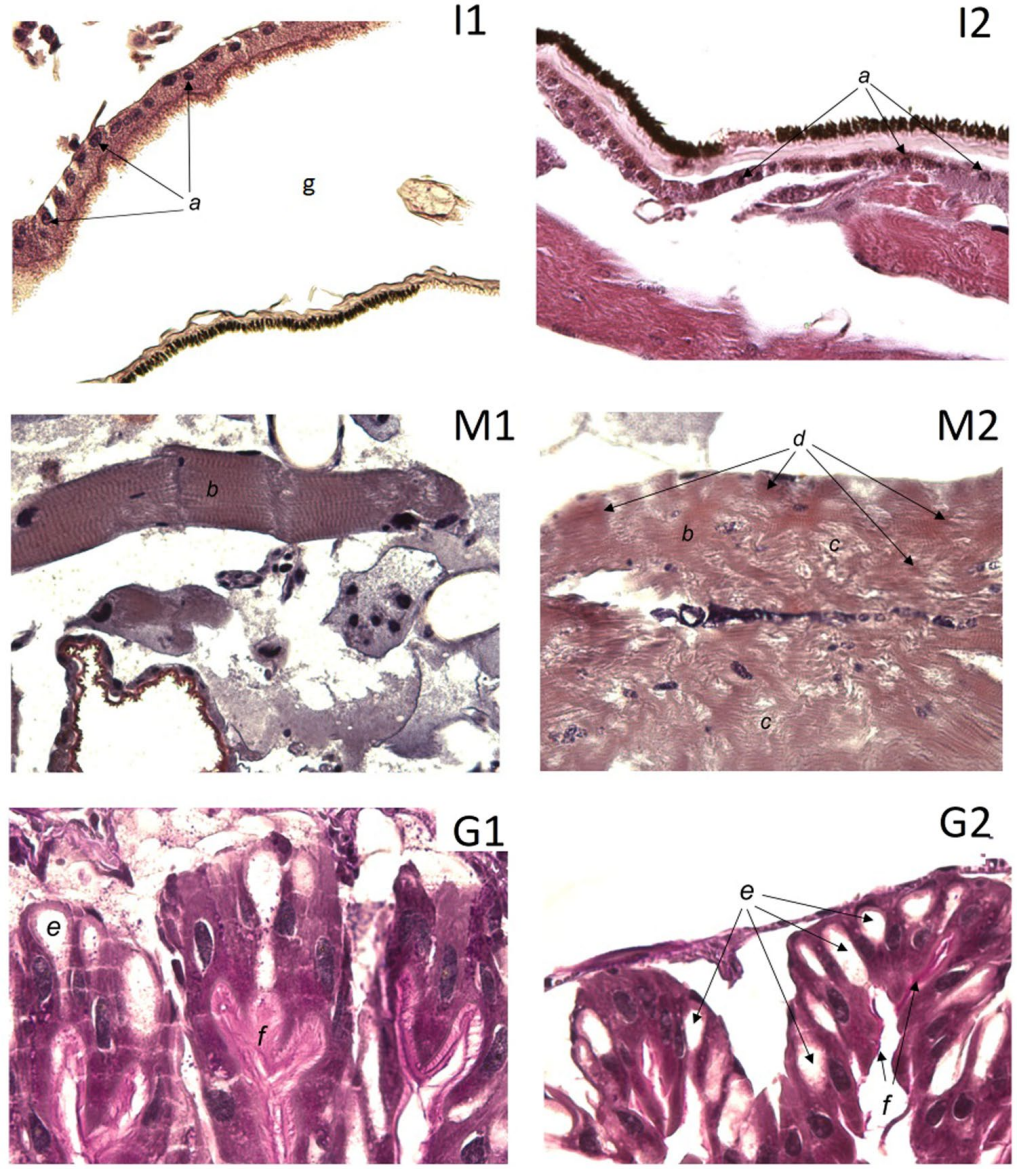
Images obtained via light microscopy examination of the histological slides of *L. dispar* larvae insect integument (I), muscle bundle (M), and midgut (G). **I1, M1, G1** — Control sample (water-treated control):a — uniform distribution of heterochromatin, well expressed nucleoli; b — ordered muscle fibers with well-defined transverse striation; e — goblet cells; f — high cilia of epithelial cells; g — intercuticular space; **I2, M2, G2** — oligoRIBO-11-treated larvae showing pronounced dystrophic destruction of tissues: a — condensation of heterochromatin around the periphery of the nucleus, absence of nucleolus; b — loss of transverse striation; c — defibration of muscle bundles; d — diffuse protein deposition; e — a large number of goblet cells; f — low cilia of epithelial cells.

Surprisingly enough, the concentration of 5.8S ribosomal RNA was not found to have decreased significantly (p > 0.05, Fig. 3) on the 12^th^ day in the surviving oligoRIBO-11-treated larvae. Either not all insects are susceptible to an applied DNA insecticide, or they are able to restore the normal concentration of 5.8S ribosomal RNA in their cells. After reviewing the data from the histological studies, which will be discussed below, in our opinion, the effectiveness of a DNA insecticide depends in large part on a larva’s proximity to molting. The closer the insect is to molting, the less effective the DNA insecticide becomes. In larvae preparing to molt, there are two layers of cuticle (old and newly formed) separated by an intercuticular space (Fig. 4,I1;g). The intercuticular space must completely fill with contact DNA insecticide before it can reach live insect tissues; as molting approaches, this space becomes substantially enlarged. This makes it more difficult for an appropriate amount of the aqueous DNA insecticide solution to fill the space enough to penetrate into the insect tissues below.

### Evidence for the development of dystrophy in tissues of oligoRIBO-11-treated *L. dispar* larvae

Using histological studies, we confirmed that the blockage of protein synthesis in *L. dispar* cells triggered by oligoRIBO-11 was responsible for significantly higher mortality. On the 6^th^ day, we used a light microscope to examine slices of stained, paraffin-embedded *L. dispar* larvae treated with oligoRIBO-11. We observed the highest proportion of pronounced dystrophic patterns in the insect integument, muscle bundles, and midgut of oligoRIBO-11-treated larvae.

Compared to that of the controls, the integument epithelium of the *L. dispar* larvae of the oligoRIBO-11 group showed signs of decreased functional activity and maturity. This was manifested by a significant increase in the nuclear-cytoplasmic ratio (due to an increase in the nucleus area and a decrease in the cytoplasmic area), which is characteristic of cells with a low degree of differentiation (in the control group the ratio was 0.28 ± 0.05, whereas in the oligoRIBO-11 group, the ratio was 0.42 ± 0.06; p < 0.01). The greatest changes were observed in nuclei, where the amount of heterochromatin increased and became condensed in large lumps along the karyolemma; in addition, the number and size of nucleoli decreased (Fig. 4,I1,I2;a). These manifestations characterize a decrease in the protein synthesis activity of cells.

Muscular bundles of oligoRIBO-11-treated *L. dispar* larvae (stained with Congo Red) showed pronounced signs of metabolic changes. A curvature of the course of muscle fibers, the loss of transverse striation, and breakage of the bundles was revealed (Fig. 4,M1,M2;b,c). Diffuse protein deposits were detected on all of the preparations, appearing as intensely pink colored fragments of various shapes and sizes, arranged in groups and also irregularly one by one in the structure of groups of muscle fibers throughout their entire length (Fig. 4,M2;d).

In addition, changes were found in the structure of the epithelial cells of the midgut of the oligoRIBO-11-treated *L. dispar* larvae. The height of the villi of the columnar cells of the midgut was markedly lower than that observed in the controls. The number of goblet cells considerably increased, which is a manifestation of a violation of the synthesis of enzymes and the absorption of nutrients (Fig. 4,G1,G2;e,f).

The changes observed in the insect integument, muscle bundles, and midgut of oligoRIBO-11-treated insects are signs of protein dystrophy caused by extensive blocking of protein synthesis.

## Discussion

This study once again showed the promise of developing DNA insecticides from antisense nucleotides. Although only half on average of the insecticide-treated insect pests died, the availability, fast speed of action, and selectivity of antisense nucleotides confirm the correctness of this research vector. Since resistance to an antisense oligonucleotide from a highly conserved region of the gene takes a long time to develop, one cannot ignore the enormous possible benefits of DNA insecticides. We can slow down insecticide resistance by using DNA insecticides based on very conservative regions of functionally important genes, such as genes encoding for ribosomal RNA. This approach is of immense value and further developments in this field may lead to safer, less expensive forestry and agriculture sustained by DNA insecticides.

The easiest way to make this approach profitable and marketable is to dramatically reduce the price of such preparations. One way to do this is to create a product composed of multiple treatments, such as a solution containing several antisense oligonucleotides that target different genes. This approach, which attacks the insect via more than one gene, is more likely to be successful and increase the mortality rate to 80–90%. Improving the efficiency of DNA synthesis could make manufacture of antisense oligonucleotides a more affordable process.

From a physical standpoint, this technology needs to be easy to apply, which can be a challenge in a natural habitat such as an oak forest. It also needs to make contact with as much surface area as possible on the body of the insect. Once it has made contact, it needs to be able to penetrate to reach the tissue vulnerable to it. With respect to application, we plan to use cold fog generators to increase the area of contact with the body of the larvae. To ensure that the insecticide penetrates to the insect tissues once it has been applied, we will use carrier molecules that increase the permeability of the insect’s chitinous outer layers. These carrier molecules should also help prevent endosomal escape of antisense oligonucleotides Much of the research concerned with creating an effective carrier has focused on hydrophilic polymers such as poly-L-lysine and polyethyleneimine^29^. Other research has been done on carriers based on dendrimers^30^. For our DNA insecticides, we are considering using poly[(organo)phosphazenes] as the carriers. The use of poly[(organo)phosphazenes] as carrier molecules for DNA fragments appears promising. They feature highly functional phosphorus-nitrogen skeletons coupled with a wide variety of possible substituents, which substantially augments the physicochemical properties of the material^31,32^. It is not surprising that poly[(organo)phosphazenes] have hold great potential as high-molecular carriers for drug and gene delivery^33^.

We would like for investors to become interested in this approach, appreciate its value, and offer to help us get it off the ground. The use of DNA insecticides seems like an excellent way to control many lepidopteran insects at the larval stage before they become breeding adults. Conventional chemical insecticides inflict great harm on nature, whereas DNA insecticides, as natural polymers, are almost completely devoid of side effects. The latter contain only water with an antisense DNA oligonucleotide dissolved in it, which is a much more natural way to control the cell of interest, in this case, an insect cell. Finding an affordable, easily applied insecticide that effectively kills pests without harming the environment is something everyone should be interested in.

The oligoRIBO-11 insecticide described here is effective, fast acting, affordable, and selective. This makes it an excellent prospective candidate for use in the development of a ready-to-use preparation based on this novel biotechnology for the protection of plants. This research and that of many others working in the field of creation of DNA insecticides and RNA preparations allows us to say very confidently that the application of nucleic acids as insecticides in agriculture and forestry is just around the corner. The search for an optimal model for the use of such preparations continues and every step taken, no matter how small, brings us closer to reaching our goal.

It is worth taking the time to look at RNA preparations and those who develop them. Enormous progress has been made by research efforts focused on the potential of insecticides developed using RNA interference (RNAi) to suppress crop pests^34^. However, retaining the selectivity that makes these preparations useful has proved difficult because of the relatively long dsRNAs in cells. When these are cleaved into numerous, very short siRNAs (21–23 nucleotides in length), they lose their selectivity, because the siRNAs have abundant direct sequence matches among the genomes of non-target organisms^35^. Moreover, it has been shown that lepidopterans lack systemic RNAi, which results in ineffective knockdown or localized knockdown (i.e., only in the midgut, where dsRNA uptake occurs), which may or may not cause mortality^36^. Most studies in the literature have reported that dsRNA ranging from 140 to 500 nucleotides in length are required to achieve successful RNAi. Different techniques such as microinjection^11^, soaking^37^, or oral feeding of artificial diet^38,39^ have been tested to try to find the best way to introduce relatively long dsRNA into an insect. Microinjection is extremely useful for introducing accurate amounts of dsRNA exactly into the chosen area. Insect ingestion of dsRNA is the most useful method for large-scale gene screening and is the main source of publications regarding RNAi control of insect pests. Transgenic plants that produce dsRNAs to be ingested by target insect pests and directed against gene function in Lepidoptera, Coleoptera, and Hemiptera pests are becoming more common^40,41^. For example, administering double-stranded RNA by feeding, as seen in *Epyphia spostvittana*^42^ and *Rhodnius prolixus*^43^, demonstrate a successful application of this technique. Soaking is convenient for research on insect cells^37^.

However, all of these delivery methods have their drawbacks. Scientists have come to the conclusion that use of GM plants should be accompanied by rigorous environmental risk assessments that take into consideration the potential for harm to non-target organisms. Clearly, our current understanding of how environmental exposure to dsRNA affects plants, animals, and insects, as well as which parameters are most likely to be responsible for off-target gene effects, is far from complete^44^. Oral feeding is hindered by the presence of dsRNases in the salivary glands and midgut of many insects, which makes the pests recalcitrant to RNAi^36^. Microinjection, while useful in the laboratory, is not a feasible method of insect pest control in a field or forest. In our opinion, among the listed delivery methods, topical treatment seems to be the most promising, since the majority of successful conventional chemical insecticides belong to the category of contact insecticides.

Insects have spent a long time evolving ways to protect themselves from predators and other bodily harm. They can run, they can fly, and they are good at camouflage. Their bodies go through a bewildering array of forms. Most pertinent to this discussion is the fact that many of them have developed armor-like outer exoskeletons that are extremely difficult to penetrate. Despite this, Wang and colleagues demonstrated that uptake of dsRNA by the entire insect body is possible by spraying dsRNA directly onto newly hatched *Ostrinia furnacalis* larvae^45^. The larvae experienced extensive mortality (40–100%), which correlated with the downregulation of the target gene expression as verified by qPCR. The results of this experiment demonstrated several important things. First, they made it clear that the insect integument can be penetrated, even when using a method as simple as spraying. Second, penetration by the dsRNAs triggered RNAi, which showed that RNAi-based pest control is possible. Finally, they confirmed the viability of this high-throughput dsRNA delivery method. Currently, research into using this application is not widespread, so the number of publications in the literature concerning the use of contact RNA preparations is limited^12^. The large relative length of the dsRNA fragments used has continued to provide obstacles to further research. We believe that RNA preparations could become as useful as DNA insecticides if the length of the RNA fragment required could be reduced, particularly in light of evidence supporting its use as a contact application. However, since they cost less to synthesize, DNA insecticides will continue to be more affordable than RNA preparations.

## Methods

### Origin of *L. dispar*

We identified and collected gypsy moth *Lymantria dispar* (Lepidoptera: Erebidae) egg masses from forested wilderness areas located near Barnaul (Altai Republic, Russia) in November 2017. In April 2018, we identified and collected gypsy moth larvae from forested wilderness areas near Simferopol (Crimea, Russia).

### Insect rearing

Under standard conditions in the lab, gypsy moth larvae were grown in Petri dishes using pubescent oak *Quercus pubescens* Wild leaves; the temperature was maintained at 25 °C. Larvae were weighed using laboratory scales with 1 mg discreteness (Axis BTU210; Axis, Gdańsk, Poland).

### Applied oligoDNA fragment sequences

Using the *L. dispar* and LdMNPV genomes found in Genbank (https://www.ncbi.nlm.nih.gov/genbank), we designed antisense oligoRIBO-11 and oligoRING fragments. The following fragment of the 5.8S ribosomal RNA gene was used: 5′-TGCGTTCGAAA-3′ (antisense strand; experimental group; oligoRIBO-11). The following sequence of the RING domain fragment of the LdMNPV IAP-3 gene was used: 5′-CGACGTGGTGGCACGGCG-3′ (antisense strand; comparative group; oligoRING). OligoDNAs were synthesized by Evrogen (Moscow, Russia).

### LdMNPV contamination exclusion

LdMNPV, which may be transmitted transovarially, is known to be widespread among gypsy moth populations^46,47^. Outbreaks that occur among populations in non-recreational forested areas tend to be controlled by naturally occurring LdMNPV epizootics^48^. To ensure that no baculovirus was present in the experimental insects, we checked the 1^st^ instar gypsy moth larvae (hatched from eggs in the lab) and those collected from a natural habitat for the LdMNPV infection using PCR, which was carried out using two oligonucleotide primers specific to the viral capsid gene p39^5,9^. All larvae were tested and found to be virus free.

### OligoDNA treatment of *L. dispar* and *G. mellonella* larvae

Before the experiment, larvae of *L. dispar* and *G. mellonella* (control, non-target organism) were randomized into groups. Larvae were then weighed to ensure that the biomass was the same for each group; the biomass among the groups was within 3% and averaged 12.3 ± 1.2 mg. Both groups (control and experimental) contained an average of 15–20 2^nd^ instar larvae. The experiment (treatment with oligoDNA fragments) was performed five times. A hand-held sprayer was used to apply a water solution with a single-stranded oligoDNA fragment (20 pmol/µL, either oligoRIBO-11 or oligoRING sequence) to larvae topically. The larvae were allowed to dry and to absorb the solution completely for 15–20 min at room temperature. Small drops of solution were collected from the surface of 20 larvae; after pulverization, we found approximately 0.3 µL solution on each larva (6 pmol of a single-stranded oligoDNA per larva).

### Quantification of *L. dispar* 5.8S rRNA

Gypsy moth larvae were ground using a pestle in liquid nitrogen in a 1.5 mL tube to prepare them for RNA extraction, which was carried out using a PureLink® RNA Mini Kit (Ambion, Life Technologies, Waltham, MA, USA), according to the manufacturer’s instructions. To produce the replicates for each treatment, three independent extractions, each with four larvae, were carried out. The quality and concentration of the extracted total RNA was assessed with NanoDrop^TM^ (Thermo Scientific, Waltham, MA, USA) and was 565.77 ± 29.5 ng/µL in different groups. For each group (control, oligoRIBO-11 or oligoRING), one extraction (with twelve larvae) was carried out; PCR was repeated three times to produce replicates for each condition. We also loaded 5 µL of the eluted volume onto a 1.5% agarose gel and ran the gel in TBE (Tris-borate-EDTA) buffer (10 V/cm) for 30 min. The quality and reproducibility of RNA extraction from the insect material was confirmed by the quantity, intensity, and pattern of the RNA bands, which were equal among all experimental groups.

For reverse transcription, the total RNA (5 µg) was annealed with RIBO-R primer (5′-GTTCGAAATGTCGATGTTC-3′) and analyzed using a RevertAid H Minus Reverse Transcriptase kit (Thermo Scientific, Waltham, MA, USA), according to the manufacturer’s instructions. The reaction was conducted at 40 °C for 60 min in Thermostat Termite (DNA Technology, Moscow, Russia). For each sample, a 0.5 µL aliquot of the obtained cDNA, with the addition of the following primers, forward 5′-AAACTATTACCCTGGACG-3′ and reverse 5′-GTTCGAAATGTCGATGTTC-3′, was used for quantitative real time PCR studies and amplification with gene specific primers to quantify the *L. dispar* 5.8S rRNA. The qPCRmix-HS SYBR (Evrogen) master mix was used according to the manufacturer’s instructions. Amplification was set up on a LightCycler® 96 instrument (Roche, Basel, Switzerland) according to the following procedure: 10 min initial denaturation at 95 °C, followed by 40 cycles with 10 s denaturation at 95 °C, 20 s annealing at 53 °C, and 12 s elongation at 72 °C. One extraction with twelve larvae was carried out for each group (Control, oligoRIBO-11, and oligoRING); PCR was repeated three times to produce replicates for each condition. As a final step, all PCR products were melted to estimate the specificity of amplification and presence of additional products.

### DNA nuclease activity analyses

Activity of intercellular DNA nucleases in tissue homogenates of target (*L. dispar* larvae) and non-target organisms (*G. mellonella* larvae*, Q. pubescens* leaves) was analyzed. A 5-mg aliquot of a target tissue was ground in 10 μL of water and then 10 μL of a target oligonucleotide at a concentration of 100 pmol/μL was added. Solutions were incubated for 0.3, 1, and 24 hours. Finally, the solutions were centrifuged for 1 min at 12,000 *g*. The resulting supernatants underwent electrophoresis on 1.8% agarose gel with standard TBE buffer and ethidium bromide (10–15 μL at a concentration of 10 mg/ml per 56 ml of 1.8% agarose gel) as a nucleic acid stain. Different concentrations of DNA fragments were used to ensure the same luminosity of oligonucleotides: 10 μL of oligoRIBO-11 at a concentration of 150 pmol/μL per electrophoresis lane and 10 μL of oligoRING at a concentration of 5 pmol/μL per electrophoresis lane.

### Histological studies

Slides were made for histological studies using gypsy moth larvae following 6 days of treatment with either 6 pmol of oligoRIBO-11 or with water (control group). Live larvae were fixed in 10% formaldehyde for 2 days, after which they were dried in isopropyl alcohol and then placed in paraffin blocks using a Logos microwave hybrid tissue processor (Milestone, Italy). A microtome (Leica RM2255, Leica Biosystems, Nussloch GmbH, Germany), was used to make 4 µm sections. The sections were stained with hematoxylin and eosin (BioVitrum, St. Petersburg, Russia), and then fixed for analysis according to the manufacturer’s instructions (Leica Biosystems Richmond, Inc., USA). Photos were taken with a Leica DM2000 microscope using the following lenses: Leica ∞/-/DFN25B N PLAN 5x/0.12, Leica ∞/-/DFN25B N PLAN 10x/0.25, and Leica ∞/0.17/DFN25D N PLAN 40x/0.65.

### Statistical analyses

The non-parametric Pearson’s chi-squared test (χ^2^) with Yates’s correction and the Mann–Whitney test were used to evaluate the significant difference between the groups’ means (STATISTICA 7 software, Palo Alto, CA, USA).

## Supplementary information

Supplementary Info File

## Data Availability

All relevant data that are not present in the paper or in the Supplementary Data are available from the authors.
