# Supplementary material for: DNA insecticide developed from the Lymantria dispar 5.8S ribosomal RNA gene provides a novel biotechnology for plant protection
Source: Sci Rep. 2019 Apr 17;9:6197. doi: 10.1038/s41598-019-42688-8 (PMC6470133; doi:10.1038/s41598-019-42688-8)
Supplement: Supplementary file 1 — Supplementary Info File [file 41598_2019_42688_MOESM1_ESM.docx]

**DNA insecticide developed from the *Lymantria dispar* 5.8S ribosomal RNA gene provides a novel biotechnology for plant protection**

Volodymyr V. Oberemok**^1^**, Kateryna V. Laikova**^2^**, Nikita V. Gal'chinsky**^1^**, Refat Z. Useinov**^1^**, Ilya A. Novikov**^1^**, Zenure Z. Temirova**^1^**, Maksym N. Shumskykh**^1*^**, Alisa M. Krasnodubets**^1^**, Anna I. Repetskaya^3^, Valeriy V. Dyadichev^4^, Iryna I. Fomochkina**^2^**, Evgenia Y. Bessalova^2^, Tatiana P. Makalish^2^, Yuri I. Gninenko^5^, Anatoly V. Kubyshkin**^2^**

**^1^**Department of Biochemistry, Taurida Academy, V.I. Vernadsky Crimean Federal University, Vernadsky Avenue 4, 295007, Simferopol, Crimea, Ukraine.

**^2^**Medical Academy named after S.I. Georgievsky, V.I. Vernadsky Crimean Federal University, Lenin Avenue 5/7, 295051, Simferopol, Crimea, Ukraine.

^3^Botanical Garden named after N.V. Bagrov, V.I. Vernadsky Crimean Federal University, Vernadsky Avenue 4, 295007, Simferopol, Crimea, Ukraine.

^4^Engineering Center, V.I. Vernadsky Crimean Federal University, Vernadsky Avenue 4, 295007, Simferopol, Crimea, Ukraine.

^5^All-Russian Research Institute for Silviculture and Mechanization of Forestry, Institutskaya Street 15, 141200, Pushkino, Russia.

Correspondence and requests for materials should be addressed to V.V.O. ([genepcr@mail.ru](mailto:genepcr@mail.ru))

**Electrophoretic gels and blots**


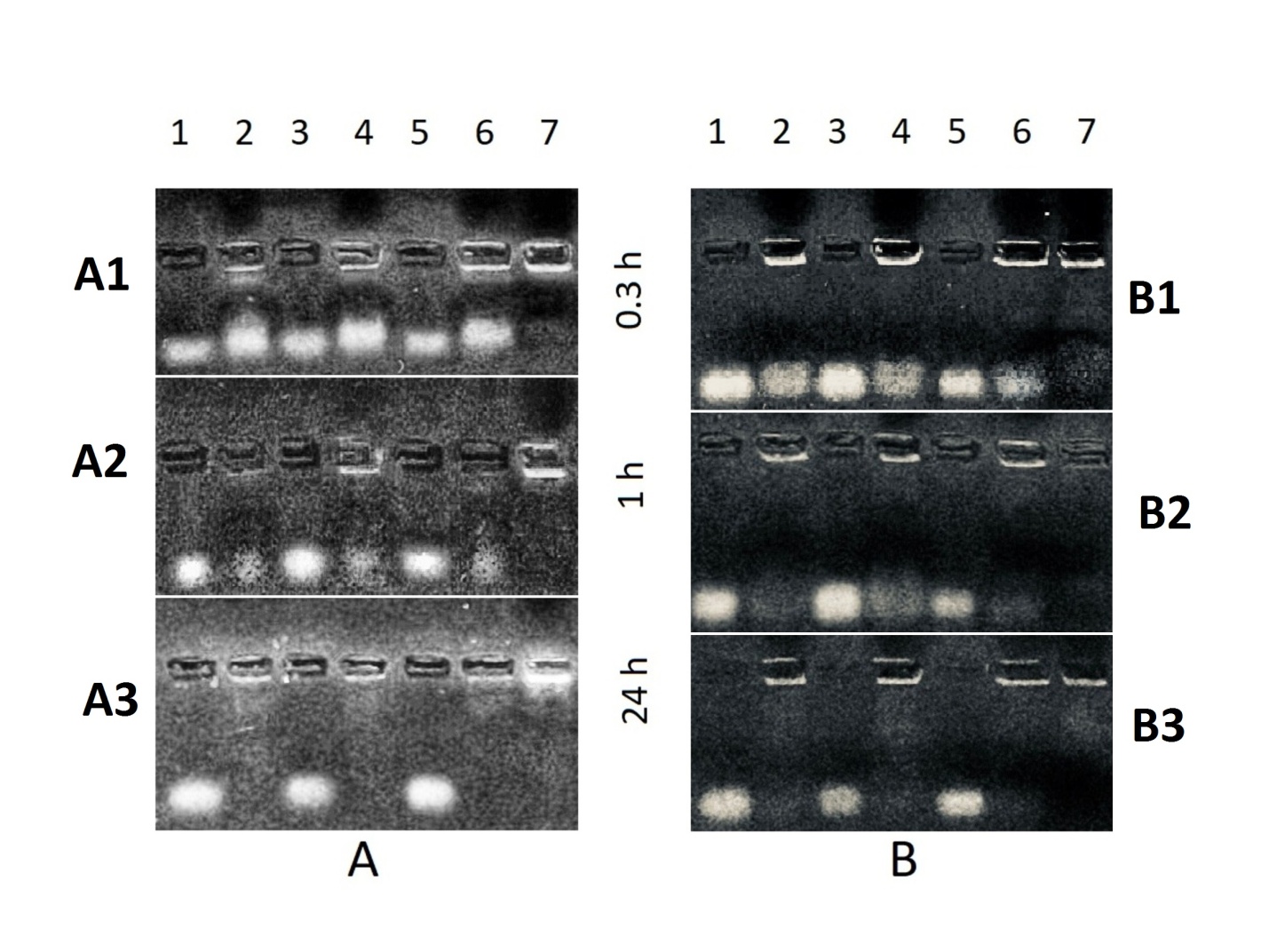


Fig. 2. Electrophoregram (1.8% agarose gel) representing the activity of intracellular nucleases of *L. dispar* (**A**) and *Q. pubescens* (**B**) after 0.3, 1, and 24 hours at 27°C: 1 – control (10 μL of oligoRIBO-11 at a concentration of 150 pmol/μL); 2 – tissue homogenate (1.5 mg of biomass per 10 μL of distilled water) + 10 μL of oligoRIBO-11 at a concentration of 150 pmol/μL; 3 – control (10 μL of oligoRING at a concentration of 5 pmol/μL); 4 – tissue homogenate (1.5 mg of biomass per 10 μL of distilled water) + 10 μL of oligoRING at a concentration of 5 pmol/μL; 5 – pure tissue homogenate (1.5 mg of biomass per 10 μL of distilled water).

^
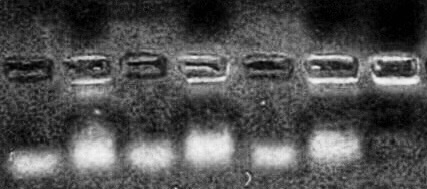
^

**A1**. Electrophoregram (1.8% agarose gel) representing the activity of intracellular nucleases of *L. dispar* after 0.3 hours at 27°C

^
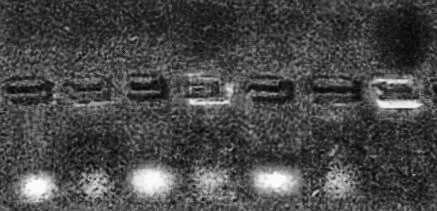
^

**A2**. Electrophoregram (1.8% agarose gel) representing the activity of intracellular nucleases of *L. dispar* after 1 hours at 27°C

^
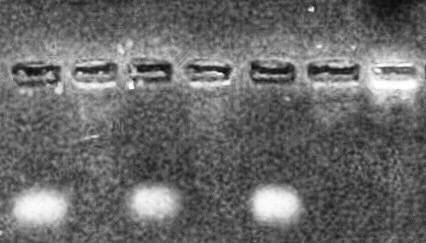
^

**A3**. Electrophoregram (1.8% agarose gel) representing the activity of intracellular nucleases of *L. dispar* after 24 hours at 27°C

^
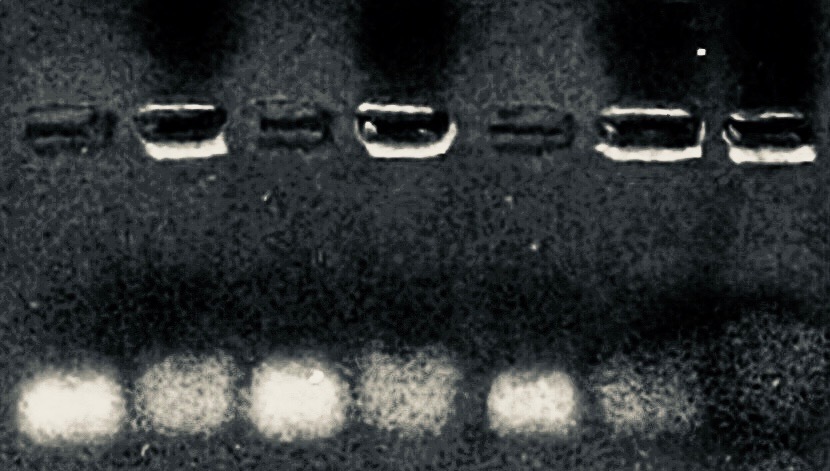
^

**B1**. Electrophoregram (1.8% agarose gel) representing the activity of intracellular nucleases of *Q. pubescens* after 0.3 hours at 27°C

^
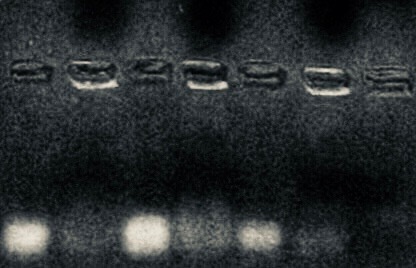
^

**B2**. Electrophoregram (1.8% agarose gel) representing the activity of intracellular nucleases of *Q. pubescens* after 1 hours at 27°C

^
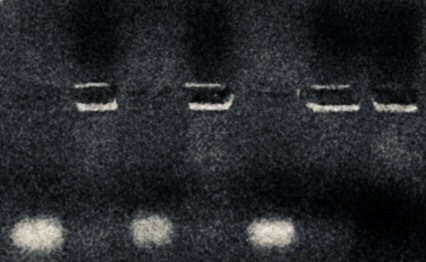
^

**B3**. Electrophoregram (1.8% agarose gel) representing the activity of intracellular nucleases of *Q. pubescens* after 24 hours at 27°C

**Competing interests**

The authors declare no competing financial and non-financial interests.
